# Supplementary material for: Clarin‐2 is essential for hearing by maintaining stereocilia integrity and function
Source: EMBO Mol Med. 2019 Aug 26;11(9):e10288. doi: 10.15252/emmm.201910288 (PMC6728604; doi:10.15252/emmm.201910288)
Supplement: Supplementary file 1 — Appendix [file EMMM-11-e10288-s001.pdf]

Clarin-2 is essential for hearing by maintaining stereocilia integrity and function

Lucy A Dunbar, Pranav Patni, Carlos Aguilar, Philomena Mburu, Laura Corns, Helena RR Wells, Sedigheh Delmaghani, Andrew Parker, Stuart Johnson, Debbie Williams, Christopher T Esapa, Michelle M Simon, Lauren Chessum, Sherylanne Newton, Joanne Dorning, Prashanthini Jeyarajan, Susan Morse, Andrea Lelli, Gemma F Codner, Thibault Peineau, Suhasini R Gopal, Kumar N Alagramam, Ronna Hertzano, Didier Dulon, Sara Wells, Frances M Williams, Christine Petit, Sally J Dawson, Steve DM Brown, Walter Marcotti, Aziz El-Amraoui, Michael R Bowl

APPENDIX

Appendix Table S1

Appendix Table S1: Exact *P*-values for ABR and DPOAE phenotyping.

				Stimulus			
**Figure**	**Comparison**	**Test**	**Age**	**8kHz**	**16kHz**	**32kHz**	**Click**
Fig 1E	$Clrn2^{clarinet/del629}$ vs $Clrn2^{+/+}$	ABR	P21	0.000000014762719	0.000000000502017	0.000000021428476	0.000000000002922
Fig 2C	$Clrn2^{clarinet/clarinet}$ vs $Clrn2^{+/+}$	ABR	P16	0.000000036463579	0.000000000031308	0.00000004454557	0.000000431700296
Fig 2D	$Clrn2^{clarinet/clarinet}$ vs $Clrn2^{+/+}$	ABR	P21	0.000000000026967	0.000000000001169	0.000000000000039	0.00000000002499
Fig 2E	$Clrn2^{clarinet/clarinet}$ vs $Clrn2^{+/+}$	ABR	P28	0.000000000009665	0.000000000000036	0.000000000144136	0.000000000000039
Fig 2F	$Clrn2^{clarinet/clarinet}$ vs $Clrn2^{+/+}$	ABR	P42	0.000000000000396	0.000000000000033	0.000000000024488	0.000000000000033
Fig 2G	$Clrn2^{clarinet/clarinet}$ vs $Clrn2^{+/+}$	DPOAE	P28	0.00111824530501	0.2266695510384	0.033988503001665	-
