# Supplementary material for: Clarin‐2 is essential for hearing by maintaining stereocilia integrity and function
Source: EMBO Mol Med. 2019 Aug 26;11(9):e10288. doi: 10.15252/emmm.201910288 (PMC6728604; doi:10.15252/emmm.201910288)
Supplement: Supplementary file 2 — Expanded View Figures PDF [file EMMM-11-e10288-s002.pdf]

Expanded View Figures

Figure EV1. *Clrn2* is essential for mammalian hearing.

A The *clarinet* mutation mapped to a ~12 Mb region on Chromosome 5 between SNPs rs6341620 and rs6192958 (Chr5:37101560-49346495, GRCm38), containing 110 genes.

B DNA sequencing identified a nucleotide transition (c.12G > A) in the *Clrn2* gene at codon 4, thus altering the wild-type (WT) sequence TGG, encoding a tryptophan (Trp), to the mutant (M) sequence TGA, encoding a premature stop codon (p.Trp4*). Electropherograms derived from a *clarinet* mutant mouse ($Clrn2^{clarinet/clarinet}$) and a wild-type colony mate ($Clrn2^{+/+}$) control showing the sequence surrounding *Clrn2* nucleotide 12 (indicated by an arrow).

C A second *Clrn2* mutant allele ($Clrn2^{del629}$) was generated employing CRIPSR/Cas9 genome editing, deleting the second coding exon of the *Clrn2* gene. Schematic representations of the genomic structure of the wild-type ($Clrn2^{+}$) and mutant ($Clrn2^{del629}$) alleles are shown. Mouse *Clrn2* consists of 3 exons, which are all in-frame to each other, spanning 10.4 kb of genomic DNA. Wild-type clarin-2 is a 232 amino acid protein, containing 4 transmembrane (TM) domains (dark grey bars). TM1 is encoded by exon 1, TM2 and part of TM3 are encoded by exon 2, and TM4 is encoded by exon 3. The ATG (translation start) and the TGA (Stop) sites are in exons 1 and 3, respectively, and the 5′ and 3′ untranslated regions are shown as black. RT–PCR of RNA extracted from cochleae of $Clrn2^{+/+}$, $Clrn2^{+/del629}$ and $Clrn2^{del629/del629}$ mice, using oligonucleotide primers designed to exon 1 (forward primer) and exon 3 (reverse primer) of the *Clrn2* gene, confirms deletion of exon 2 in the mutant mice and identifies aberrant splicing of exon 1 to exon 3, which are in-frame. As such, the $Clrn2^{del629/del629}$ transcript has the potential to generate a shorter clarin-2 isoform, but this would be missing two of the four transmembrane domains that define the tetraspan clarin-2 protein.

D Averaged ABR click waveforms for $Clrn2^{+/+}$, $Clrn2^{clarinet/+}$, $Clrn2^{del629/+}$, $Clrn2^{clarinet/del629}$ and $Clrn2^{clarinet/clarinet}$ mice at P21. Arrows indicate the sound intensity at which the auditory threshold was called.

 © 2019 The Authors

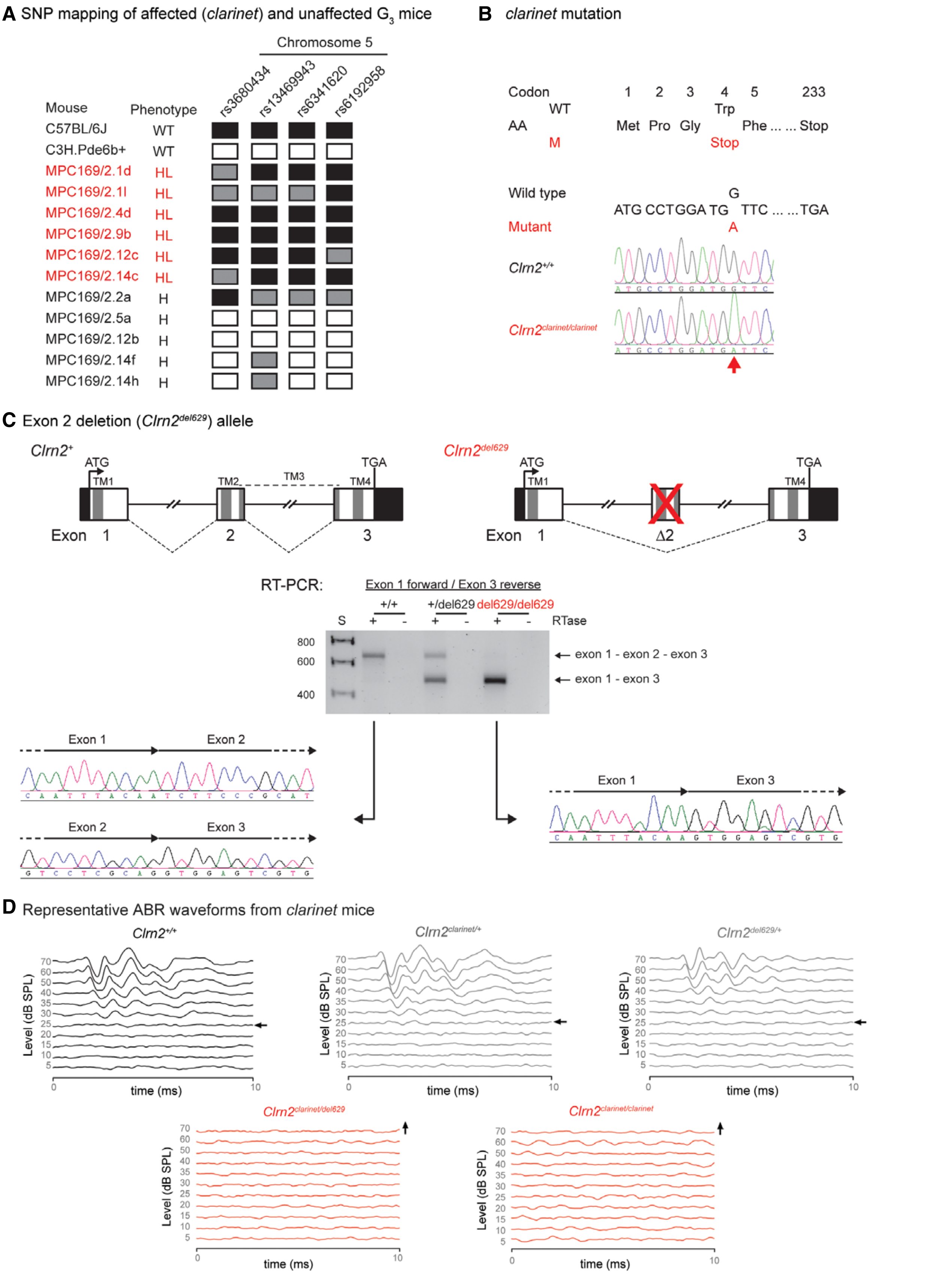
A SNP mapping of affected (clarinet) and unaffected G3 mice
Chromosome 5
rs3680434
rs13469943
rs6341620
rs6192958
Mouse
Phenotype
C57BL/6J WT
C3H.Pde6b+ WT
MPC169/2.1d HL
MPC169/2.1l HL
MPC169/2.4d HL
MPC169/2.9b HL
MPC169/2.12c HL
MPC169/2.14c HL
MPC169/2.2a H
MPC169/2.5a H
MPC169/2.12b H
MPC169/2.14f H
MPC169/2.14h H
B clarinet mutation
Codon 1 2 3 4 5 233
WT
AA Met Pro Gly Trp Phe ... ... Stop
M Stop
Wild type G
ATG CCTGGA TG TTC ... ...TGA
Mutant A
Clrn2+/+
Clrn2clarinet/clarinet
C Exon 2 deletion (Clrn2del629) allele
Clrn2+
ATG TGA TM1 TM2 TM3 TM4
Exon 1 2 3
Clrn2del629
Exon 1 Δ2 3
RT-PCR: Exon 1 forward / Exon 3 reverse
+/+ +/del629 del629/del629
S + - + - + - RTase
800
600
400
exon 1 - exon 2 - exon 3
exon 1 - exon 3
Exon 1 Exon 2
Exon 2 Exon 3
Exon 1 Exon 3
D Representative ABR waveforms from clarinet mice
Clrn2+/+
Clrn2clarinet/+
Clrn2del629/+
Clrn2clarinet/del629
Clrn2clarinet/clarinet
Level (dB SPL)
time (ms)

Figure EV1.

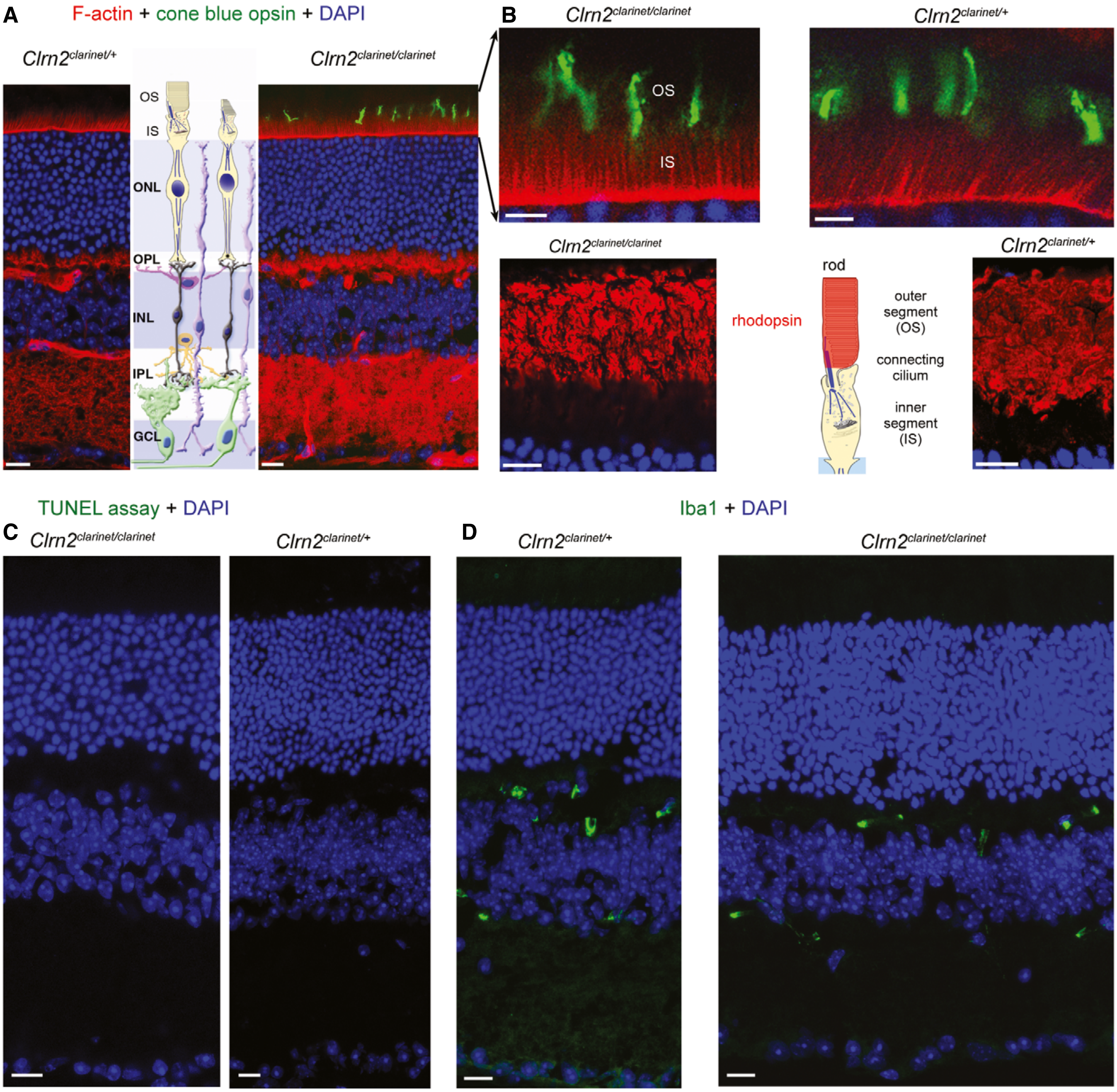

Figure EV2. Absence of a retinal phenotype in *Clrn2*$^{clarinet/clarinet}$ mice at 7 months.

A–D Representative retinal cryosections of *Clrn2*$^{clarinet/+}$ and *Clrn2*$^{clarinet/clarinet}$ mice at 7 months. Phalloidin and DAPI staining show the retina have a normal thickness and architecture, with a normal photoreceptor cells layer organization (A). Focusing on photoreceptor cells, we found no difference in the distribution of cone opsin or rhodopsin, which were confined to the outer segments of cones and rods, respectively, in *Clrn2*$^{clarinet/+}$ and *Clrn2*$^{clarinet/clarinet}$ mice (B). The absence of morphological or functional abnormalities in *Clrn2*$^{clarinet/clarinet}$ mice is consistent with absence of TUNEL-positive nuclei (C) and normal distribution of Iba-1, a gliosis marker (D). IS/OS, inner segment and outer segment; ONL, outer nuclear layer; OPL, outer plexiform layer; INL, inner nuclear layer; IPL, inner plexiform layer; GCL, ganglion cell layer. Scale bars, 20 µm.

 © 2019 The Authors

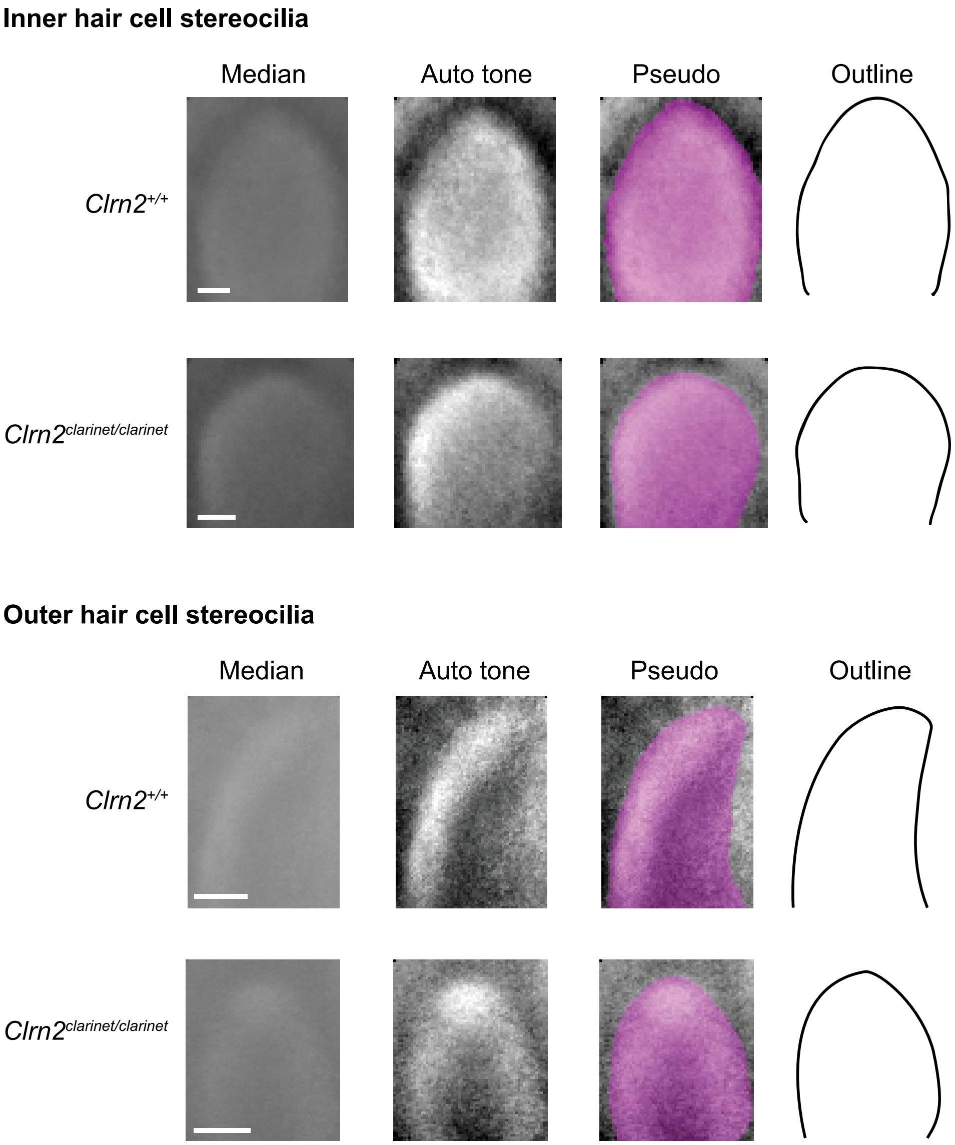

Figure EV3. Median Z-projections of IHC and OHC stereocilia tips from the second tallest row.

A pseudo-stack comprising of ≥ 80 images of individual stereocilia tips extracted from scanning electron micrographs prepared from P8 wild-type and *clarinet* mutant mice. The Adobe Photoshop "Auto tone" function was used to increase the contrast of each median projection, followed by pseudo-colouring to highlight the edges of the projected "averaged" stereocilia tips. These image adjustments allow an accurate outline of each median projection to be drawn. For both inner and outer hair cells, the tips of the stereocilia from the second tallest row of *Clrn2*$^{clarinet/clarinet}$ mice appear more rounded than those of their wild-type (*Clrn2*$^{+/+}$) littermates.

© 2019 The Authors

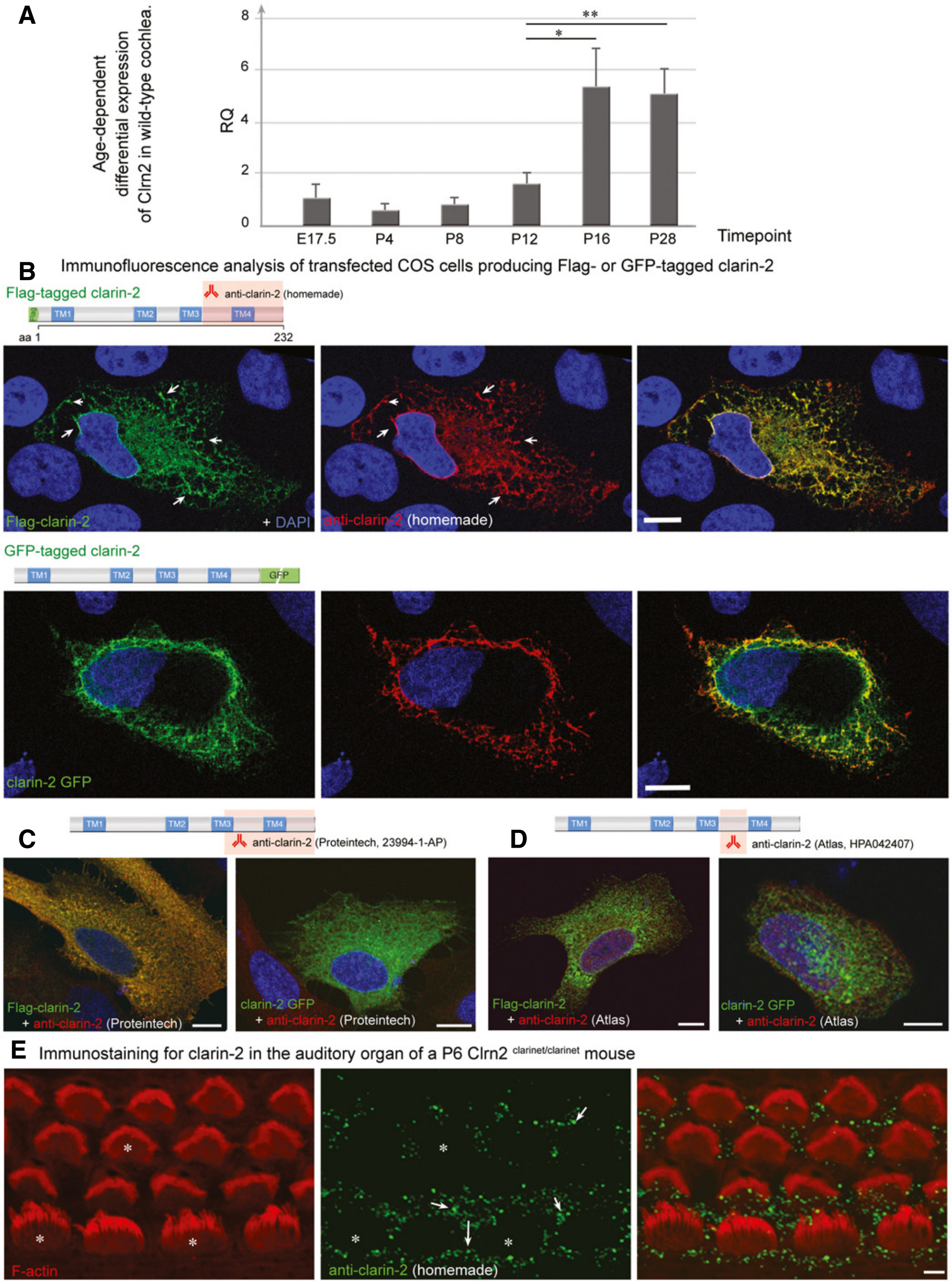

Figure EV4. Age-dependent differential expression of *Clrn2* in wild-type cochlea, and tests of anti-Clrn2 antibodies.

A RT–PCR analysis. To assess the temporal expression of *Clrn2* in the cochlea, cochlear RNA was extracted from wild-type mice at several perinatal and early postnatal timepoints and utilized for qRT–PCR studies to determine the abundance of *Clrn2* transcripts. Values were calculated relative to expression level at P4. Expression is constant during embryonic and early postnatal timepoints (E17.5 to P12), then increases ~3-fold between P12 and P16. RQ: relative quantification (arbitrary units). For each timepoint, the data shown are mean ± SD of 5 biological replicates. $*P < 0.05$, $**P < 0.01$, one-way ANOVA.

B–D Clarin-2 expression and test of anti-clarin-2 antibodies. Transfected HeLa cells producing FLAG- or GFP-tagged clarin-2 (green) were labelled by the anti-clarin-2 antibodies (green): homemade (B), commercial Proteintech (23994-1-AP) (C) or Atlas (HPA042407) (D). Only the homemade antibody clearly labelled the two over-expressed clarin-2 fusion proteins (overlapping immunostaining in yellow–some highlighted by arrows, B). Conversely, none of the commercial antibodies could detect the GFP-tagged clarin-2, as visualized by the lack of yellow staining in the left panels in (C) (Proteintech) and (D) (Atlas).

E Immunostaining using anti-clarin-2 homemade antibody showed no specific staining in the F-actin-labelled hair cells (asterisks). The white arrows indicate the presence of non-specific immunostaining over the supporting cells.

Data information: Scale bars: 10 µm (B–D), 2 µm (E).

 © 2019 The Authors

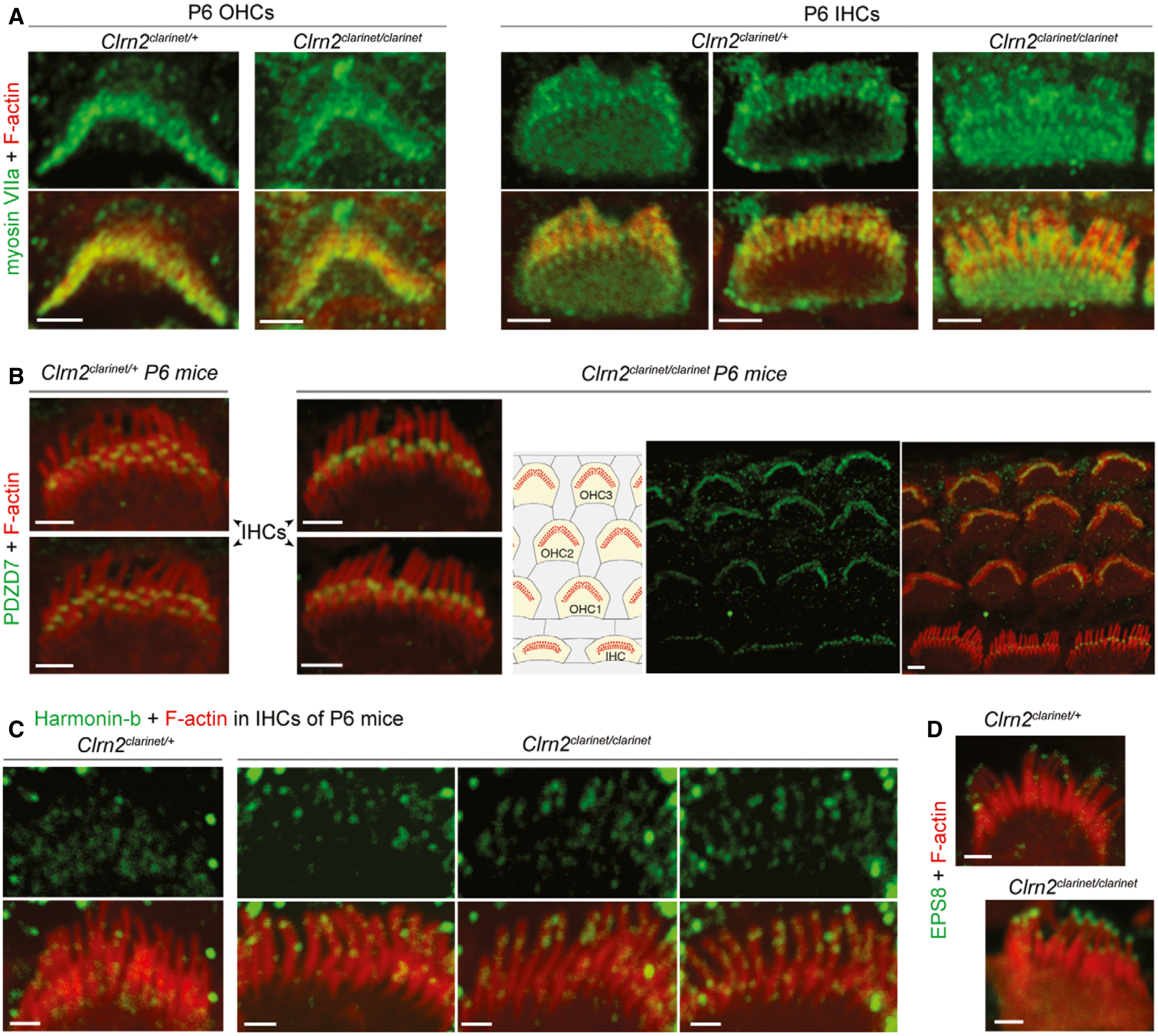

Figure EV5. Distribution of hair bundle proteins in *clarinet* mice.

A–D Confocal images of whole-mount preparations of cochlear sensory epithelia from *Clrn2*$^{clarinet/+}$ and *Clrn2*$^{clarinet/clarinet}$ P6 mice immunostained for the Usher 1B protein myosin VIIa (green in A) and actin (red), PDZD7 (green in B) and actin (red). The PDZD7 immunostaining is normally restricted to the base of stereocilia in both *Clrn2*$^{clarinet/+}$ and *Clrn2*$^{clarinet/clarinet}$ mice. (C) Examples of IHCs from *Clrn2*$^{clarinet/+}$ and *Clrn2*$^{clarinet/clarinet}$ P6 mice, showing the change in harmonin-b localization in the absence of Clrn2. (D) EPS8 immunostaining (green) illustrating the protein enrichment at the tips of actin-labelled stereocilia (red) in both *Clrn2*$^{clarinet/+}$ and *Clrn2*$^{clarinet/clarinet}$ IHCs. Scale bars, 2 μm.
