# Supplementary material for: Clarin‐2 is essential for hearing by maintaining stereocilia integrity and function
Source: EMBO Mol Med. 2019 Aug 26;11(9):e10288. doi: 10.15252/emmm.201910288 (PMC6728604; doi:10.15252/emmm.201910288)
Supplement: Supplementary file 3 — Table EV1 [file EMMM-11-e10288-s003.docx]

# **Table EV1. 20 most highly associated SNPs in the *CLRN2* region**

| SNP | Position | Effect allele | Frequency | INFO score | BETA | SE of | P-Value | Consequence | Impact | GENE symbol |
| --- | --- | --- | --- | --- | --- | --- | --- | --- | --- | --- |
| rs35414371 | 17530692 | T | 0.867 | 0.998 | -0.013 | 0.002 | 1.60E-11 | Intergenic variant | MODIFIER | - |
| rs13147559 | 17524570 | C | 0.867 | 1.000* | -0.013 | 0.002 | 1.70E-11 | Missense variant | MODERATE | CLRN2 |
| rs34859220 | 17521797 | G | 0.866 | 0.998 | -0.013 | 0.002 | 2.30E-11 | Intron variant | MODIFIER | CLRN2 |
| rs36027619 | 17525356 | C | 0.867 | 1.000 | -0.013 | 0.002 | 2.40E-11 | Intron variant | MODIFIER | CLRN2 |
| rs13148153 | 17517558 | C | 0.866 | 0.996 | -0.013 | 0.002 | 2.80E-11 | Intron variant | MODIFIER | CLRN2 |
| rs6449307 | 17522511 | G | 0.578 | 0.998 | -0.008 | 0.001 | 1.40E-08 | Intron variant | MODIFIER | CLRN2 |
| rs1551092 | 17517861 | A | 0.422 | 0.996 | 0.008 | 0.001 | 1.60E-08 | Intron variant | MODIFIER | CLRN2 |
| rs773007268 | 17527566 | AT | 0.576 | 0.997 | -0.007 | 0.001 | 2.20E-08 | Intron variant | MODIFIER | CLRN2 |
| rs2443041 | 17526995 | A | 0.425 | 0.999 | 0.007 | 0.001 | 2.60E-08 | Intron variant | MODIFIER | CLRN2 |
| rs10648467 | 17521238 | C | 0.420 | 0.993 | 0.007 | 0.001 | 2.70E-08 | Intron variant | MODIFIER | CLRN2 |
| rs2034494 | 17526270 | T | 0.425 | 0.999 | 0.007 | 0.001 | 2.70E-08 | Intron variant | MODIFIER | CLRN2 |
| rs62295121 | 17531916 | G | 0.506 | 0.992 | -0.007 | 0.001 | 6.10E-08 | Intergenic variant | MODIFIER | - |
| rs34571936 | 17520383 | T | 0.467 | 0.973 | 0.007 | 0.001 | 9.30E-08 | Intron variant | MODIFIER | CLRN2 |
| 4:17532145_CT_C | 17532145 | CT | 0.496 | 0.981 | 0.007 | 0.001 | 2.70E-07 |  |  |  |
| rs17458406 | 17510236 | T | 0.866 | 0.975 | -0.009 | 0.002 | 2.40E-06 | Intron variant | MODIFIER | QDPR |
| rs142647241 | 17551144 | C | 0.989 | 0.790 | -0.033 | 0.007 | 3.30E-06 | Regulatory region variant | MODIFIER | - |
| 4:17536714_AT_A | 17536714 | AT | 0.527 | 0.996 | -0.006 | 0.001 | 3.70E-06 |  |  |  |
| rs13117648 | 17539024 | A | 0.564 | 0.981 | -0.006 | 0.001 | 3.70E-06 | Intergenic variant | MODIFIER | - |
| rs5016868 | 17539016 | A | 0.564 | 0.981 | -0.006 | 0.001 | 4.20E-06 | Intergenic variant | MODIFIER | - |
| rs147634912 | 17536537 | A | 0.564 | 0.991 | -0.006 | 0.001 | 5.10E-06 | Intergenic variant | MODIFIER | - |

Position; position on genome build hg19; Frequency; effect allele frequency; BETA, effect size from BOLT-LMM approximation to infinitesimal mixed model; SE, standard error of effect size; INFO score, compound of imputation score and missingness, * indicates genotyped SNP; Consequence, consequence of variant at that position; Impact, impact of variant on function predicted by Ensembl variant effect predictor. The Bonferroni corrected significance threshold based on the effective number of independent SNPs within this region is 0.0009.
