# Supplementary material for: Clarin‐2 is essential for hearing by maintaining stereocilia integrity and function
Source: EMBO Mol Med. 2019 Aug 26;11(9):e10288. doi: 10.15252/emmm.201910288 (PMC6728604; doi:10.15252/emmm.201910288)
Supplement: Supplementary file 4 — Table EV2 [file EMMM-11-e10288-s004.docx]

**Table EV2. Sequences of oligonucleotides utilised for the generation of the *Clrn2^del629^* allele.**

| **Oligonucleotide name** | **Sequence** |
| --- | --- |
| *Clrn2* CRISPR sgRNA protospacer, 5’_1 (PAM) | ACTCTGCCAAGCCAATGCCT (TGG) |
| *Clrn2* CRISPR sgRNA protospacer, 5’_2 (PAM) | AGCCAATGCCTTGGGCTTAT (GGG) |
| *Clrn2* CRISPR sgRNA protospacer, 3’_1 (PAM) | AGGGCACTTTTATTGCAGTC (TGG) |
| *Clrn2* CRISPR sgRNA protospacer, 3’_2 (PAM) | AGACTCAAAAGGAAGCTAAA (GGG) |
| *Clrn2* CRISPR genotyping primer (forward) | ACGAGCTCACTCAACCCCTAA |
| *Clrn2* CRISPR genotyping primer (reverse) | TGAAGGTCCGCCTTTGACCA |
| *Clrn2* CRISPR copy counting ddPCR primer (forward) | GCTCTGGTCAGCATGGGTT |
| *Clrn2* CRISPR copy counting ddPCR primer (reverse) | GGGCCATTGACTGCTCTGTA |
| CRISPR copy counting ddPCR probe | TTGCTATTCTCAACATCATTCAGGTCCC |
